# The MexMAGIC population reveals the genetic architecture of traits exhibiting clinal variation in Mexican native maize

**DOI:** 10.1111/nph.71501

**Published:** 2026-09-04

**Authors:** Sergio Perez‐Limón, Ana Laura Alonso‐Nieves, M. Rosario Ramírez‐Flores, G. Carolina Cintora‐Martínez, M. Nancy Salazar‐Vidal, Jorge Vladimir Torres‐Rodriguez, Jessica Carcaño‐Macías, Melanie G. Perryman, Oliver H. S. Paulson, Jagdeep S. Sidhu, Peng Yu, Victor Llaca, Kevin A. Fengler, Forrest Li, Daniel E. Runcie, Jeffrey Ross‐Ibarra, C. Stewart Gillmor, Rubén Rellán‐Álvarez, Ruairidh J. H. Sawers

**Affiliations:** ^1^ Department of Plant Science The Pennsylvania State University University Park PA 16802 USA; ^2^ Unidad de Genómica Avanzada Cinvestav, Irapuato Guanajuato 36824 México; ^3^ Departamento de Fitomejoramiento Universidad Autónoma Agraria Antonio Narro Calzada Antonio Narro 1923, Colonia Buenavista Saltillo Coahuila 25315 México; ^4^ Departamento de Biotecnología y Bioquímica Centro de Investigación y de Estudios Avanzados (CINVESTAV‐IPN) Irapuato Guanajuato 36821 México; ^5^ Division of Plant Science and Technology University of Missouri Columbia MO 65211 USA; ^6^ The Traits Company Research Triangle Park NC 27713 USA; ^7^ Department of Plant Genetics, TUM School of Life Sciences Technical University of Munich Freising 85354 Germany; ^8^ Genome Center of Excellence Corteva Agriscience Johnston 50131 IA USA; ^9^ Department of Evolution and Ecology University of California Davis CA 95616 USA; ^10^ Department of Plant Sciences University of California Davis CA 95616 USA; ^11^ Department of Molecular and Structural Biochemistry, N.C. Plant Sciences Initiative North Carolina State University Raleigh NC 27695 USA

**Keywords:** cline, flowering time, genetic architecture, MAGIC, Mexican maize, tassel branch number

## Abstract

- Defining the genetic basis of local adaptation is a key goal of evolutionary biology and crop improvement. Theory predicts that when selective pressures follow differences in the environment, a cline will be established. Clines can be exploited to uncover adaptive variation by association of alleles with the environment. However, monotonic phenotypic change over a cline is not necessarily mirrored in the behavior of genetic variants and population structure can further complicate analysis.
- To study genetic and phenotypic variation across the environment, we developed a multi‐parent advanced generation inter‐cross (MAGIC) population using eight Mexican native maize (*Zea mays* L. ssp. *mays*) varieties sourced from distinct agroecological zones. We evaluated the population in a common garden in Mexico and mapped tassel branching and flowering time, two traits that exhibit clinal variation.
- Variation in tassel branching was dominated by a single QTL with allele effects aligning to a negative elevational cline. By contrast, allele effects associated with 11 identified flowering time QTL were not consistently correlated with any one source environmental factor.
- Our observations support the prediction that genotype‐environment association will be strongest under simple genetic architecture, although, even then, analysis in native populations may be confounded by population structure.

## Graphical abstract

The MexMAGIC population mixes native maize varieties from across Mexico to study functional genetic diversity.

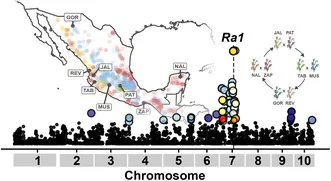

## Resumen

- Uno de los objetivos clave en la biología evolutiva y el mejoramiento genético de cultivos es descifrar la base genética de la adaptación local. Trabajos teóricos han predicho que cambios graduales en las presiones de selección a lo largo de un ambiente heterogéneo pueden resultar en clinas. El estudio de la base genética de las clinas puede ayudar en detectar variación genética adaptativa a través de la asociación entre variación alélica y ambiental, sin embargo, los cambios monotónicos observados en un fenotipo no necesariamente son el resultado de cambios monotónicos graduales en frecuencias alélicas. Además, la presencia de estructural poblacional puede complicar la interpretación de asociaciones entre genotipo y ambiente.
- Con el objetivo de estudiar la relación entre variación genética y fenotípica entre ambientes, desarrollamos una población multiparental MAGIC (multi‐parent advanced generation inter‐cross, por sus siglas en inglés) generada con la contribución de ocho variedades de Maíces Nativos Mexicanos (*Zea mays* L. ssp. *mays*) procedentes de distintas zonas agroecológicas. La población MexMAGIC fue evaluada en un experimento de jardín común en México, y se evaluaron el número de ramas en la espiga y tiempo de floración, dos fenotipos que muestran variación clinal.
- La variación fenotípica en el número de ramas en la espiga está determinada principalmente por un único QTL, cuyos efectos alélicos siguen un gradiente altitudinal negativo. Por el contrario, los efectos alélicos asociados a los 11 QTL identificados para el tiempo de floración no mostraron una correlación consistente con ningún factor ambiental específico.
- Nuestras observaciones respaldan la predicción de que la asociación genotipo‐ambiente es más fuerte cuando existe una arquitectura genética sencilla; no obstante, incluso en estos casos, el análisis en poblaciones nativas puede verse afectado por la estructura poblacional.

## Introduction

A cline is a pattern of monotonic change in a biological character over an environmental gradient (Huxley, 1938; Savolainen *et al*., 2013; Lotterhos, 2023). Population genetic theory predicts that clinal variation will arise across continuous populations when selective pressures follow differences in the environment (Huxley, 1938; Haldane, 1948; Fisher, 1950). Many empirical examples of clinal variation have been identified over both geographic axes (e.g. latitude and elevation) and gradients of specific environmental factors (e.g. temperature, precipitation). While not all phenotypic variation is adaptive, clines can be exploited to generate hypotheses regarding the adaptive value of a trait with respect to a given environmental pressure (Stinchcombe *et al*., 2004; Koski & Ashman, 2015). Such inferences can be greatly strengthened by assessing repeatability across parallel clines, although this itself makes assumptions about contingency and reproducibility in trait selection.

With the availability of large‐scale genomic datasets linked to collections of georeferenced biological diversity, cline theory can be used to support the direct identification of adaptive alleles through genotype‐environment association (GEA; Lasky *et al*., 2023). GEA is trait agnostic and relies only on correlation between allele frequency and source environment. The genome can be scanned for GEAs in an environmental Genome Wide Association (GWA), providing an attractive opportunity to mine broad diversity for useful variation without the need for logistically challenging and costly phenotypic evaluation (e.g. Key *et al*., 2018; Mao *et al*., 2019; Li *et al*., 2025). Although environmental GWA has great potential, it rests on assumptions that may, or may not, hold for a given study system. Isolation by distance along a cline will tend to limit geneflow and establish population structure that is aligned with, and thereby confounding, the genetic signals underlying adaptation (Ahrens *et al*., 2021; Lasky *et al*., 2023). As such, control of population structure in an environmental GWA, while necessary, risks eliminating the very signals that are the focus of the study. More fundamentally, GEA assumes that gradual monotonic change in adaptive traits over an environmental cline is reflected by similar behavior in allele frequency at the causative loci. The validity of this assumption will be greatly impacted by the genetic architecture of underlying adaptive traits, that is, the number of causative loci, the size and specificity of their effects, and their interactions with each other and with the environment. As genetic architecture becomes more complex, the effect of any given allele may be small, and indeed even neutral over sections of the landscape. In addition, individuals equally well adapted to a particular locality might harbor distinct, but functionally equivalent, complements of adaptive alleles. Under such a scenario, the frequency of any given allele may be only weakly correlated with the environment even if the emergent patterns of local adaptation at the whole genotype level are strong.

The crop maize (*Zea mays* L. ssp. *mays*) is a compelling study system for the characterization of links between genetic and environmental variation (e.g. Li *et al*., 2025; McLaughlin *et al*., 2025), being distributed over a broad environmental range, and the focus of extensive fundamental and applied research. Maize was domesticated from Balsas teosinte (*Zea mays* L. ssp. *parviglumis* Iltis & Doebley; hereafter, *parviglumis*) by indigenous peoples at least 9000 yr ago in the basin of the Balsas River in Mexico (Matsuoka *et al*., 2002; Piperno *et al*., 2007). Mexico remains a center of maize diversity, with 59 documented native varieties (Sanchez *et al*., 2000). Although each variety has its own character and associated culinary and cultural importance (Louette *et al*., 1997; Ortega‐Paczka, 2003; Cleveland & Soleri, 2007; Mercer & Perales, 2019), overlapping distribution and inter‐varietal hybridization mean that Mexican maize as a whole can be considered a continuous, if structured, population (Ortega‐Paczka, 2003; Campbell *et al*., 2025). Mexican native maize is cultivated from the tropical jungles of Chiapas to the highland semideserts of Chihuahua, from sea level to *c*. 3400 m above sea level (m asl) (Ruiz Corral *et al*., 2008; www.biodiversidad.gob.mx). Previous characterization has documented local adaptation in Mexican maize associated with morphological and physiological clines, including elevational variation in flowering time, tassel branching, stem pigmentation and pubescence, and latitudinal variation in seminal root number (Wellhausen *et al*., 1952; Wellhausen *et al*., 2013; Eagles & Lothrop, 1994; Sanchez *et al*., 2000; Mercer *et al*., 2008; Romero Navarro *et al*., 2017; Mercer & Perales, 2019; Janzen *et al*., 2022; Perez‐Limón *et al*., 2022; Yu *et al*., 2024).

The genetic basis of local adaptation in Mexican maize has been investigated through comparative genomics, and both phenotypic and environmental GWA (Romero Navarro *et al*., 2017; Wang *et al*., 2021; Li *et al*., 2025). To complement such work and address the difficulty presented by strong environmentally correlated population structure, we previously developed a biparental linkage mapping population using a highland Mexican maize variety and a reference inbred line (Janzen *et al*., 2022; Perez‐Limón *et al*., 2022). Here, we extend this approach with the development of a multi‐parent advanced generation inter‐cross population (MexMAGIC) that retains some of the power of our previous linkage population while sampling broader diversity and breaking the structure of the native population (Scott *et al*., 2020). We present genetic mapping of tassel branching and flowering time, two traits that differ in the complexity of their genetic architecture, but that have both been reported to vary over an elevational cline (Romero Navarro *et al*., 2017; Janzen *et al*., 2022). Mapping was conducted over two seasons in a single common garden environment in Mexico. The MexMAGIC population captures haplotypes sampled from across an elevational range (from 13 to 2597 m asl), allowing us to compare allele effects in the common garden to an expectation derived from the location of the source of the allele along the established cline. We discuss our results in the context of the genetic architecture of clinal variation and their implications for GEA approaches. In addition, this report provides a template for use of the MexMAGIC as a resource in future research.

## Materials and Methods

### Selection of MexMAGIC founders

To capture haplotypes sourced from across the environmental range of Mexican native maize, eight accessions were selected corresponding to varieties placed in different climatic‐adaptation groups in a previous analysis of 42 native varieties (Eagles & Lothrop, 1994; Ruiz Corral *et al*., 2008). Three varieties (REV, TAB, and ZAP) were represented by inbred (S_6_) derivatives of outbred accessions (Ames 30532‐MR18; Ames 32 914‐MR21; Ames 30 536‐MR23; Supporting Information Table S1; Chia *et al*., 2012; Venkatesh *et al*., 2015), and the other five varieties (GOR, JAL, MUS, NAL, and PAL) by outbred open‐pollinated varieties (OPVs) obtained from the International Center for Maize and Wheat Improvement (CIMMyT) seed bank (CIMMYTMA‐007937, CIMMYTMA‐002246, CIMMYTMA‐005582, CIMMYTMA‐000815, CIMMYTMA‐002233; Table S1). The accession of the highland variety PAL was selected because of previous genomic (Vielle‐Calzada *et al*., 2009) and genetic (Perez‐Limón *et al*., 2022) characterization. Accessions of JAL and NAL were selected as members of a previously characterized core diversity set (Wellhausen *et al*., 1952; Reif *et al*., 2006). Subsequent classification has questioned whether the accession Nayarit 6 (CIMMYTMA‐002246) is representative of the Jala variety, although the passport data are considered accurate (Denise Costich & Cristian Zavalla Espinosa, unpublished observation). The varieties GOR and MUS were selected to represent the unique environments of Chihuahua and Michoacán, respectively.

### Environmental analysis of the Mexican diversity panel

To provide context for the selected MexMAGIC founders, we defined a broader Mexican diversity panel (CIMMYT panel) from a collection of georeferenced and genotyped native maize varieties previously characterized by CIMMYT (Romero Navarro *et al*., 2017). After filtering for Mexican origin (latitude ranging from 14.38 to 30.64; longitude ranging from −111.12 to −86.92; three Guatemalan accessions were retained), availability of genotype data, and unambiguous variety assignment, we obtained a set of 1379 accessions (Table S2). Georeference data were used to associate climate data for each accession from the WorldClim Database (Hijmans *et al*., 2005) and the Global Soil Dataset for use in Earth System Models (GSDE; Shangguan *et al*., 2014) using {R/raster::extract} (Hijmans, 2023) as previously described (Lasky *et al*., 2015; McLaughlin *et al*., 2024). Environmental descriptors selected for further analysis were annual and seasonal mean temperature and precipitation, mean temperature of the coldest month, soil pH, and seasonal average photoperiod. To calculate seasonal means, the growing season was approximated as May to October for all varieties and climatic zones. Soil pH was calculated as the mean of pH‐H_2_O, pH‐KCl and pH CaCl_2_ descriptors from GSDE. Photoperiod was calculated from latitude using {R/geosphere::daylength} (Hijmans, 2022). Accessions were assigned to varieties using the GrinGlobal database (https://www.grin‐global.org) and subsequently matched by variety name to climatic‐adaptation groups based on a previously published classification (Eagles & Lothrop, 1994; Ruiz Corral *et al*., 2008) and plotted on the map of Mexico using {R/rworldmap::map.polygon} (South, 2011). Environmental principal component (PC) analysis was performed on the MexMAGIC founders and Mexican diversity panel accessions with the eight environmental descriptors and elevation as variables, using {R/ade4::dudi.pca} (Dray & Dufour, 2007). Variables were centered and scaled for analysis.

### Generation of MexMAGIC families

Crosses among the eight selected MexMAGIC founders (five outbred varieties and three S_6_ inbred lines) were attempted following a diallel scheme, and a set of four successful F_1_ combinations was selected to cover all eight parents. Subsequently, four‐way crosses were made using a single individual from each F_1_ to capture a single haplotype per founder using the ‘chain’ scheme A to B, B to C, C to D, and D to A. An equal number of seeds was advanced from each four‐way ear for a round of eight‐way crosses using multiple individuals, followed by three generations of random intermating and three generations of inbreeding. For simplicity, the final families were designated Intermated3 Selfed3 (I_3_S_3_). The I_3_S_3_ MexMAGIC were test‐crossed to the inbred line NC358 for field evaluation. NC358 is part of the temperate‐adapted all‐tropical group of lines developed by North Carolina State University (Nelson *et al*., 2016) and was selected as well supported by genomic resources and for good pollen production in the Mexican nursery fields used for testcross seed production. Additional details of population generation are provided in Methods S1 and Figs S1–S3.

### Determination of MexMAGIC founder haplotypes

To determine the eight founder haplotypes entering the MexMAGIC, we generated whole‐genome sequence (WGS) at *c*. 33× coverage for the four F_1_ individuals, two S_6_ inbred founders (Tabloncillo and Reventador) and OPV accessions of Jala and Palomero Toluqueño (Table S1), as well as generating full‐genome assemblies of S_6_ inbred founders Zapalote Chico and Tabloncillo (www.maizegdb.org/HiLo_project). To identify genetic variants segregating in the population, we used the GATK Best Practices workflow for Germline short variant discovery (Poplin *et al*., 2018) for both WGS and full genome assemblies. Initial quality assessment and preprocessing of WGS data were performed under default parameters using tools described in https://bioinformaticsworkbook.org/dataAnalysis/VariantCalling/gatk‐dnaseq‐best‐practices‐workflow.html#gsc.tab=0. Short reads were aligned to the B73 genome v.4 (Jiao *et al*., 2017) using bwa (Li & Durbin, 2009). A first round of variant discovery from BAM files was performed using {gatk::HaplotypeCaller}. Identified variants were filtered by quality values and used as a training set to recalibrate the BAM files using {gatk::BaseRecalibrator}. A second round of variant discovery was performed in the recalibrated BAM files using {gatk::HaplotypeCaller}, resulting in a GVCF. The full genome assemblies of Zapalote Chico and Tabloncillo were aligned to the B73 v4 reference genome using the {AnchorWave::genoAli} function (Song *et al*., 2022) with ‐IV parameter to detect inversions and other options as default, as suggested in https://github.com/baoxingsong/AnchorWave. The resulting MAF files were converted to GVCF using the {TASSEL5::MAFToGVCFPlugin} plugin (Bradbury *et al*., 2007) with default parameters according to https://github.com/baoxingsong/AnchorWave/blob/master/doc/GATK.md.

The GVCFs generated for the WGS samples and full genome assemblies were combined into a single database using {gatk::GenomicsDBImport}, and a joint calling on the full cohort was performed with {gatk::GenotypeGVCFs}. The resulting VCFs were hard filtered using default parameters as described in https://gatk.broadinstitute.org/hc/en‐us/articles/360035890471‐Hard‐filtering‐germline‐short‐variant. SNPs with missing information were removed, resulting in a final VCF containing *c*. 16 million SNPs distributed across the 10 maize chromosomes. VCFs were transformed into HAPMAP format using TASSEL5 (Bradbury *et al*., 2007) and passed to a custom script in {R} (R Core Team, 2022) that implemented a triplet approach comparing parents and their F1 to estimate the allele contributed by each of the two parents at sites heterozygous in the F1 cross. For the crosses TAB × MUS, GOR × REV and NAL × ZAP, one of the two parents was represented by an S_6_ partial inbred. For the Cross JAL × PAT, both parents were OPVs. Sites that could not be resolved were dropped, yielding a final set of *c*. 8 million pseudo‐homozygous SNPs for the eight founders of the MexMAGIC population.

### PacBio HiFi long‐read sequencing (LRS) sequencing of Palomero Toluqueño, Zapalote Chico and Tabloncillo

PacBio HiFi‐grade genomic DNA (gDNA) was isolated from *c*. 0.5 g of frozen young leaf tissue from single plants using the NucleoBond® HMW DNA kit (Cat. 740160.20; Macherey‐Nagel, Allentown, PA, USA) following the manufacturer's instructions. Native gDNA was sheared to a 10–20 kb fragment size distribution using a Diagenode Megaruptor 3, and fragment size was assessed with the Agilent Femto Pulse System. Fragments shorter than 10 kb were removed using the PippinHT system (Sage Science; Beverly, MA, USA). Sequencing libraries were prepared with the SMRTbell Express Template Prep Kit 2.0 (Pacific Biosciences, San Diego, CA, USA) and sequenced using Chemistry 2.0 on the PacBio Sequel II platform, following standard protocols. Data were collected from two SMRT Cells per sample in 30‐h movies. HiFi reads shorter than 5 kb were filtered in silico after sequencing. For the Tabloncillo accession, 4438 228 reads totaling 72.93 Gb (*c*. 34× coverage) with an average read length of 16.4 kb were obtained. For Zapalote Chico, 4745 054 reads totaling 71 Gb with an average read length of 15.9 kb were collected, and for Palomero de Jalisco, 5529 410 reads totaling 63.7 Gb with an average read length of 11.8 kb were obtained. For Palomero Toluqueño, 38× with a read N50 of 14.5 kb was obtained. See also www.maizegdb.org/HiLo_project.

### Bionano DLS optical genome mapping

Ultra‐high molecular weight (uHMW) DNA extraction and optical genome mapping using the Bionano Direct Label and Stain (DLS) Chemistry were performed as described in Hufford *et al*. (2013). The resulting molecule datasets were filtered to retain only molecules larger than a minimum length threshold needed to reach a total raw molecule coverage of *c*. 150–180×. For Tabloncillo, 998 133 molecules ≥350 kb were collected, totaling 491 Gb. For Zapalote Chico, 1179 310 molecules ≥300 kb were obtained, totaling 498 Gb. Bionano Access v.1.7.2 and Bionano Tools v.3.7 were used to process, visualize, assemble and scaffold maps. Zapalote Chico assembly produced 86 maps with an N50 length of 89 Mb and total length of 2147 Mb. The assembly of Tabloncillo led to 69 maps with an N50 length of 99.74 and 2217 Mbp of total length.

### Genotypic analysis of MexMAGIC families

Ten seeds each of 221 MexMAGIC families, I_3_S_3_ families and the MexMAGIC founders were germinated in the glasshouse and tissue was collected for genotypic analysis. For MexMAGIC families, tissue of 10 individuals was pooled. For MexMAGIC founders, individuals were analyzed separately. Leaf tissue was pulverized to a fine powder using a mortar and pestle in liquid N_2_. DNA was extracted from frozen tissue using the DNeasy Plant Kit (Qiagen). A total of 240 samples were analyzed using the Illumina MaizeLD BeadChip (https://www.illumina.com/documents/products/datasheets/datasheet_maize_snp50.pdf) by the Genomic Services Laboratory at the Advanced Genomics Unit, Irapuato, Mexico. Polymorphic sites with a call rate > 95% were used for further analysis, yielding *c*. 20 000 markers.

### Generation of a MexMAGIC Practical Haplotype Graph

To impute founder haplotypes onto the chip‐genotyped MexMAGIC families, we built a custom Practical Haplotype Graph (PHG; Bradbury *et al*., 2022). The PHG was built with 1000 intervals (100 per chromosome) and populated using the 8 founder haplotypes and the B73 reference (Jiao *et al*., 2017). The founder haplotype HAPMAP file was transformed to VCF using TASSEL5 and then converted to BAM using {gatk::SimulateReadsForVariants} and finally to GVCF using {gatk::HaplotypeCaller}. The resulting GVCFs were used to populate the PHG and impute onto the MexMAGIC families genotyped with the Illumina MaizeLD BeadChip. Imputation increased the number of single nucleotide polymorphisms (SNPs) per family from *c*. 10 000 to *c*. 6 M SNPs. The imputed set was filtered by minor allele frequency consistent with the expected frequency across the founders, generating a dataset of *c*. 3.5 M SNPs. To estimate the relative contribution of each founder to the population, the founder assigned to each range in each family was extracted using the {r/rphg} package (Monier *et al*., 2024). The genome‐wide founder and missing data proportion were estimated as the count of ranges assigned to the founder across the complete population (200 families) divided by the total number of ranges (1000 ranges per family).

### Population structure of MexMAGIC families

The filtered set of 10 191 BeadChip SNPs for MexMAGIC families and founder parents was converted to numeric format using TASSEL and missing marker data were imputed using {R/rrBLUP::A.mat} (Endelman, 2011) under default parameters. Population structure was estimated as the first five principal coordinates (PCoAs) obtained from multidimensional scaling of the genotype matrix using {R/stats::cmdscale}(Figs S4–S6). The relationship between PCo2 and source elevation for the founder parents was modeled using {R/stats::loess} with a span of 0.4 determined by inspection, and theoretical elevations for the MexMAGIC families obtained from the loess fit using {R/stats::predict}.

### Parental assignment across the genomes of MexMAGIC families

To characterize the genetic mosaic of the MexMAGIC families, a subset of 1 M random SNPs was selected with stratification by chromosome using the {R/rsample::initial_split} function (Frick *et al*., 2024). To estimate the genetic position of markers in the MexMAGIC dataset, a monotonic spline was trained on the genetic map of the IBM (Intermated B73 × Mo17) maize population (Lee *et al*., 2002; Balint‐Kurti *et al*., 2007) using the {R/scam} package (Pya & Wood, 2015) and was used to interpolate the genetic position of MexMAGIC SNPs anchored to the B73 physical map. The genetic and physical map, and the founder and MexMAGIC families genotypes were combined into a cross object to be used by the {R/qtl2} package. Redundant SNPs were identified with the {qtl2::find_dup_markers} function and dropped, generating a final dataset of 17 030 markers. The founder conditional genotype probabilities at every marker were estimated with the {qtl2::calc_genoprob} function, and the founder genotype with maximum marginal probability was estimated with the {qtl2::maxmarg} function.

### Field evaluation of MexMAGIC families

MexMAGIC families were evaluated as test‐crosses to the inbred line NC350 in the summers of 2021 and 2022 in Ameca, Jalisco, Mexico (20.575024, −104.038857; 1235 m asl). Two complete blocks were evaluated in 2021 and 2022, consisting of 190 and 175 families, respectively (see Supporting Information). The distribution of the families was completely randomized within each block. Fifteen seeds per family were sown in 2 m long rows. Plants were grown under conventional agronomic conditions. A commercial hybrid adapted to the region was planted as a check across the field (Figs S7, S8).

### Evaluation of an elevational cline in tassel branch number

Tassel branch number (TBN), source elevation and genotype data for 52 Mexican native maize accessions (HiLo panel) were obtained from Janzen *et al*. (2022) (Fig. S9). The published sampling was stratified by elevation, with paired ‘high’ and ‘low’ accessions selected along a latitudinal gradient from northern to southern Mexico. TBN data were summarized as the median of values reported for highland and lowland common gardens. The published genotypic data were filtered by minor allele frequency > 0.1 and missing information < 0.2, to 4060 SNPs using TASSEL5 (Bradbury *et al*., 2007) and converted to numeric format. Missing marker data were imputed using {R/rrBLUP::A.mat} (Endelman, 2011) under default parameters. Population structure for the Janzen *et al*. (2022) data were estimated as the first five principal coordinates (PCoAs) obtained from multidimensional scaling of the genotype matrix using {R/stats::cmdscale} (Fig. S10). The relationship between source elevation and TBN was investigated using {R/stats::lm} to fit the fixed‐effect models:

(Eqn 1) TBN~μ+Elevation+ε

(Eqn 2) TBN~μ+PCo1+PCo2+PCo3+PCo4+PCo5+Elevation+ε

Although significant in the first model (Eqn 1; *P* < 0.001), the elevation effect on TBN did not survive inclusion of population PCo1‐5 in the second model (Eqn 2; type II ANOVA elevation *P* = 0.99), reflecting the structuring of the panel by elevation and the correlation between PCo1 and elevation (Fig. S11).

### Mapping in the MexMAGIC population

To map trait variation in the MexMAGIC founders, we used two different mapping methodologies in the {R/qtl2} package (Broman *et al*., 2019): SNP‐association and 8‐allele QTL mapping. Both methods use a mixed linear model to model the phenotypic variation as a function of the genetic variation in the population, with the addition of a kinship matrix to account for residual population structure. The SNP‐association method collapses the marginal founder probabilities into biallelic allele probabilities and performs the association at two different genetic levels; the 8‐allele QTL model directly uses the marginal founder conditional probabilities, performing the association at eight different levels corresponding to the eight founders. Kinship was estimated using a ‘leave one chromosome out’ (LOCO) function {qtl2::calc_kinship()}. Founder conditional genotype probabilities were converted to allele probabilities using the {qtl2::genoprob_to_alleleprob()} function. The SNP‐association was performed using {qtl2::scan1snps()}. The 8‐allele mapping was performed using {qtl2::scan1}. To establish a significance threshold, 1000 permutations were run using the function {qtl2::scan1perm}, considering a significance threshold (alpha) of 0.1. Significant QTLs were identified with the function {qtl2::find_peaks} and confidence intervals defined as a drop of 2 in LOD value from the QTL peak (Broman *et al*., 2003). To obtain an estimation and confidence interval of founder allele effects, the marginal genotype probabilities at the lead SNP were extracted using {ql2::pull_genoprobpos}. The genotype probabilities, kinship matrix, and phenotypic data were used as input for the {qtl2::fit1} function to fit a single‐QTL model using a mixed linear model and kinship matrix to control for residual structure. The estimated allele effects and SE were extracted from the QTL object for analysis and visualization. Results of SNP‐association and 8‐allele mapping were presented as Manhattan plots using {R/ggplot2} package for R (Wickham, 2016).

### Test of environmental clines in the MexMAGIC population

To test for alignment between allele effects at candidate SNPs and elevation, a mixed linear model was fitted using {R/lme4} and {R/lmertest} (Bates *et al*., 2015; Kuznetsova *et al*., 2017):

(Eqn 3) $$\text{trait}\sim \mu +\mathrm{PCo}1+\mathrm{PCo}2+\mathrm{PCo}3+\mathrm{PCo}4+\mathrm{PCo}5+\text{elevation}+\left(1|\text{parent}\right)+\varepsilon$$

We modeled trait variation as a function of the fixed effects of the first five genetic PCos, source elevation, and the random effect of the hard call on founder genotype (Eqn 3). The marginal genotype probabilities at a given candidate SNP were extracted using the {ql2::pull_genoprobpos} function, and the hard call for every family (genotype with the maximum marginal probability given a probability of at least 0.9) was estimated with the {qtl2::maxmarg}. The hard call for a family with a maximum marginal probability < 0.9 was considered as missing. To make use of observations without a hard call, the elevation was estimated as the dot product between the marginal genotype probabilities and the founder‐allele source elevation. The significance of the model was extracted from the resulting object of the {lmerTest::anova()}function.

### Genome‐wide association using environmental descriptors

Environmental GWAS was performed using genotyping‐by‐sequencing data (GBS) from the 1379 accessions of the Mexican HiLo panel and 8 environmental descriptors (Table S2) using a previously described pipeline (Li *et al*., 2025). Briefly, environmental descriptors were rank‐inverse‐normal transformed and GBS SNPs were filtered to a minor allele frequency > 0.01 for a total of 343 205 markers. To perform multi‐trait GWAS on multiple environmental variables, the package megalmm (Runcie *et al*., 2021) was first run with a kinship matrix generated from the GBS data and all transformed environmental variables, and the de‐correlated genetic and residual covariance matrices included as structured error terms in an EMMAX ANOVA model implemented in the package jointgwas. To identify potential candidate genes from MEGALMM environmental GWAS analysis, the SNP‐based evidence of association (*P*‐values) was collapsed into gene‐based evidence using MAGMA (de Leeuw *et al*., 2015). After an FDR correction (Storey *et al*., 2025), only the top 1% significant genes were kept.

## Results

### MexMAGIC founders were sourced from across the range of Mexican maize cultivation

To characterize the genetic architecture of trait variation in Mexican native maize, we generated a multi‐parent advanced generation inter‐cross (MAGIC) population (Scott *et al*., 2020) using eight founder haplotypes sourced from across the Mexican environment (MexMAGIC; hereafter, we refer to the founder varieties by three‐letter identifiers given in Table 1). The eight founders were selected to sample varieties across previously described climatic‐adaptation groups (Eagles & Lothrop, 1994; Ruiz Corral *et al*., 2008) and showed good coverage of both the geographical (Fig. 1a) and environmental (Fig. 1b; Tables S2, S3) range of Mexican maize distribution (CIMMYT panel; Romero Navarro *et al*., 2017). For pragmatic reasons, we used derived inbred (S_6_) stocks as founders where available (Chia *et al*., 2012; Venkatesh *et al*., 2015), although it remained necessary to source five of the eight founders from outbred open pollinated varieties (OPVs) to sample the environments of interest. We restricted the second generation in our breeding scheme to four single F_1_ individuals, each derived from a distinct hybrid cross, ensuring that each founder donated only a single haplotype to the final population (Figs 1c, S1–S3). After subsequent rounds of intermating (see Materials and Methods section), we generated > 500 MexMAGIC partially inbred (S_3_) families. Here, we present genetic and phenotypic characterization of a first cohort of 200 families. As partial inbreds derived largely from outbred varieties, the MexMAGIC families exhibit low *per se* vigor, and, in consequence, were test‐crossed to the inbred line NC358 for field evaluation.

**Table 1:** Source of the maize (*Zea mays* L. ssp. *mays*) MexMAGIC founders.

Founder	Label	Latitude	Longitude	Elevation (m asl)
Reventador	REV	21.95	−105.22	13
Nal‐Tel	NAL	20.85	−88.52	30
Zapalote Chico	ZAP	16.32	−95.24	50
Jala	JAL	21.11	−104.45	1098
Tabloncillo	TAB	19.97	−104.27	1355
Gordo	GOR	29.65	−108.25	2141
Mushito	MUS	19.42	−101.56	2483
Palomero Toluqueño	PAT	19.29	−99.57	2597

**Fig. 1:**
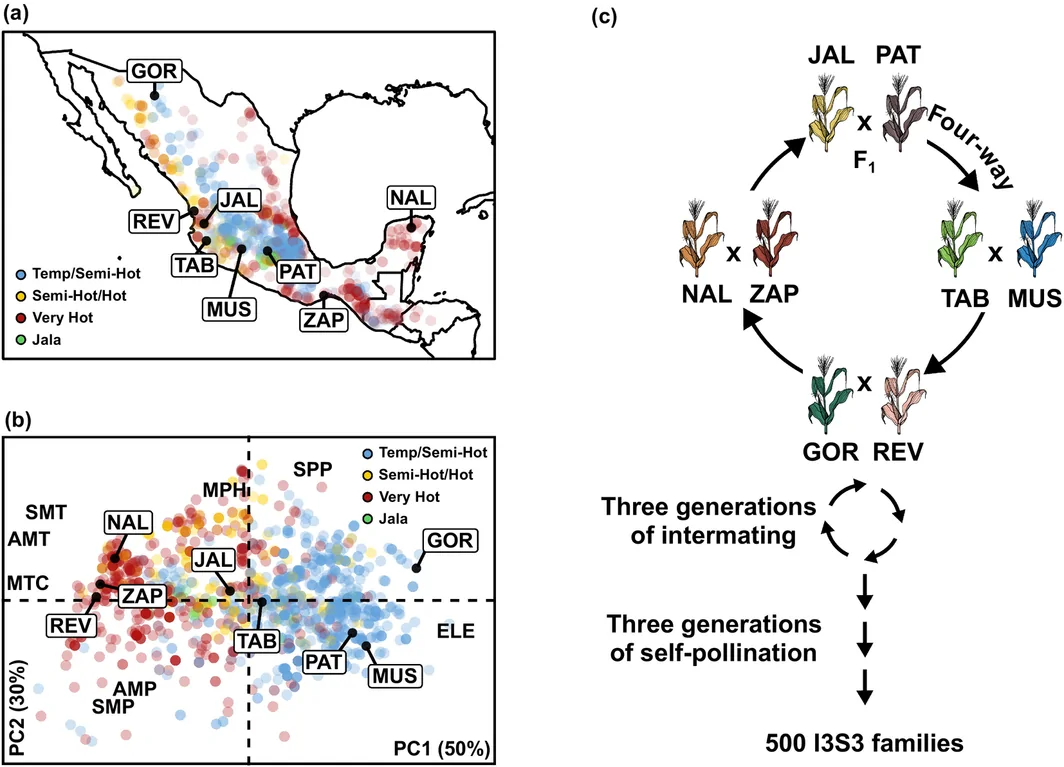
Founder selection and design of the MexMAGIC population. (a) Source of the eight founder maize (*Zea mays* L. ssp. *mays*) varieties (boxed three‐letter identifiers) in the context of a broader Mexican diversity panel (Romero Navarro *et al*., 2017) labeled at the variety level by previously assigned agroecological environments (Ruiz Corral *et al*., 2008). (b) Environmental principal component (PC) analysis of MexMAGIC founders (boxed three letter identifiers) and Mexican diversity panel based on elevation (ELE), seasonal photoperiod (SPP), annual mean temperature (AMT), seasonal mean temperature (SMT), annual mean precipitation (AMP), seasonal mean precipitation (SMP), mean temperature of the coldest month (MTC) and mean soil pH (MPH). Biplot shows PC1 (explaining 50% variance) and PC2 (explaining 30% variance). Position of environmental descriptor labels indicates relative importance and sign of loading. Variety points colored as in (a). (c) Crossing scheme for generation of the MexMAGIC from eight founder parents.

### The genomes of the MexMAGIC families are mosaics of the founder parents

To determine the eight MexMAGIC founder haplotypes, we generated *c*. 30× whole‐genome sequence (WGS) data for the four founding F_1_ individuals, two of the three inbred founders (TAB, REV), the OPV JAL parent, and sibling individuals sampled from the founder OPVs (Table S1). We had previously generated WGS data for the OPV PAT founder (Gonzalez‐Segovia *et al*., 2019). Similarly, further WGS data were available for the three inbred founders (TAB, REV, ZAP) from an earlier maize HapMap study (Chia *et al*., 2012; Venkatesh *et al*., 2015). While working on the MexMAGIC, we also generated complete genome assemblies for TAB and ZAP inbreds, along with a haploid assembly for an individual sampled from the same PAT OPV accession used in the MexMAGIC population (www.maizegdb.org/HiLo_project). For each F_1_ genotype, we used a triplet approach to phase heterozygous sites with respect to the founder parents and determine the founder haplotypes (Fig. S4A). Across the eight founder haplotypes, we identified 14 million polymorphic sites, anchored to the B73 reference genome, that were assembled into a haplotype graph for subsequent analysis of the derived MexMAGIC families (Bradbury *et al*., 2022).

A set of 200 MexMAGIC families (Fig. S4B) were genotyped using a 50 k SNP array. After filtering, we obtained *c*. 10 k polymorphic SNPs (Table S4). Principal coordinate (PCo) analysis showed the MexMAGIC families to be broadly scattered across the genetic space defined by the eight founders (Fig. S4C; Table S5). There were several families that showed outlying loading on PCo1 (11 > 2SDs above the mean) that was distinct from other families and the founders (Figs S4C, S9), although, when analyzed at the chromosome level, loading on PCo1 fell within the range defined by the founders for all families (Fig. S6). We speculate that structural variation might be driving outlying PCo patterns, although further data and analyses will be required to fully address this possibility. For the present, given that we had already generated phenotypic data, we chose to retain all 200 MexMAGIC families for subsequent analysis. PCo2 nicely separated the founders by source elevation with good coverage of MexMAGIC families over the range (Fig. S4C,D; Table S5). The SNPs identified with the genotyping array were used as a scaffold to impute the founder WGS SNPs into the families. After imputation and filtering for missing data, 8 million polymorphic SNPs were obtained per family. After filtering for redundancy, a final set of 17 030 markers was obtained. SNP data were used to estimate the probability of each founder haplotype across the genome for each family (Fig. S4E). Where unambiguous, the most probable founder at any position in the genome was used to make a hard call and infer gene‐level allele origin for subsequent analyses (Table S6). On average, we observed 180 crossovers per family, and a median recombination segment length of 5.77 MB (Table S4). Given the challenges of accurately estimating a *de novo* genetic map from a relatively small number of MAGIC families, we interpolated genetic positions for our B73‐anchored markers using the available IBM (intermated B73 × Mo17) high‐resolution map (Lee *et al*., 2002; Balint‐Kurti *et al*., 2007). The genome‐wide allele frequencies are balanced around expectation, ranging from 0.11 for GOR and PAT to 0.13 for ZAP, and a proportion of missing data of 0.08. (Supporting Information).

### Major kernel color loci segregate in the MexMAGIC population

To assess QTL mapping in the MexMAGIC population, we characterized two highly heritable kernel pigment traits associated with well‐defined large‐effect loci (Chen *et al*., 2024). We first mapped the accumulation of red flavonoid‐derived phlobaphenes in the maternal pericarp tissue, known to be regulated by the *myb*‐like transcription factor *P1* (Grotewold *et al*., 1994). In the MexMAGIC, red kernel pigmentation was unambiguously donated by the REV parent with red pigmentation only seen to segregate in the REV OPV and the derived S6 inbred used as a founder (Fig. 2a). We then characterized yellow carotenoid pigments in the endosperm, known to be produced by a phytoene synthase encoded by the *Y1* gene (Buckner *et al*., 1996; Palaisa *et al*., 2004). Yellow pigmentation segregated in NAL, PAT and MUS OPVs (Fig. 2a). The JAL × PAT F1 ear did not segregate yellow kernels, indicating that PAT did not donate an active *Y1* allele to the population (Fig. S1). Unfortunately, pigmentation in the F1 ears TAB × MUS and NAL × ZAP was not recorded, although three of the four 4‐way ears segregated yellow kernels, indicating that both MUS and NAL had donated *Y1* alleles. These segregation patterns provided a clear expectation of not only the loci but also the parental allele effects anticipated to underlie kernel pigmentation in the MexMAGIC.

**Fig. 2:**
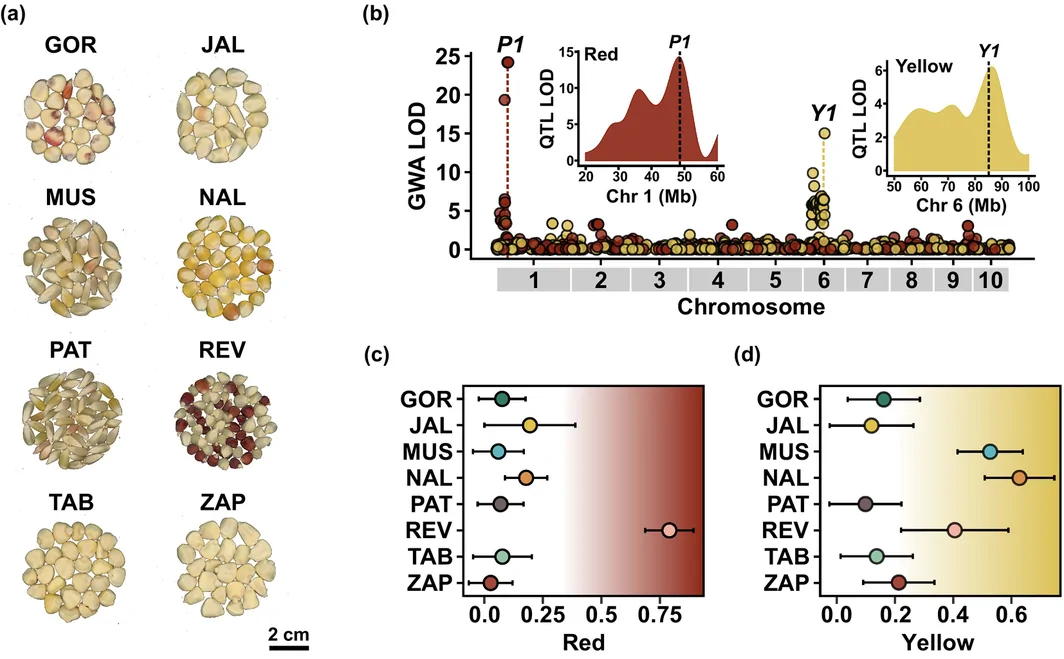
Classical loci are associated with variation in pericarp and aleurone pigmentation in the MexMAGIC. (a) Kernels of the eight MexMAGIC maize (*Zea mays* L. ssp. *mays*) founder accessions. The inbred founders REV, TAB, and ZAP are represented here by related open‐pollinated accessions. (b) Main plot shows genome‐wide association (GWA) support (LOD) for association with red pericarp (red points) or yellow aleurone (yellow points) pigmentation across the 10 maize chromosomes. All markers with LOD > 3 are shown. A reduced random sampling of lower‐supported markers is shown. The physical position of the candidate genes *P1* and *Y1* is indicated by a dashed vertical line. Inset plots show smoothed LOD support for 8‐allele QTL models for red and yellow pigmentation around *P1* and *Y1*, respectively. (c, d) Estimated founder allele effects at markers linked to *P1* and *Y1* for red and yellow pigmentation, respectively. Pigmentation (red or yellow) was scored per family as a binary trait, with 0 as non‐pigmented and 1 as pigmented. Points show BLUPs of QTL effects for each founder. Bars show ± SE.

We scored red and yellow kernel pigmentation as binary traits (presence/absence) across 196 MexMAGIC families (Table S7). Red pigmented families were removed from the yellow analysis due to the ambiguity of scoring pigmentation in the aleurone underlying the red pericarp. Families that showed segregating pigmentation (in the case of red, at the ear level due to segregation in maternal genotype; in the case of yellow, at the kernel level, due to segregation of the endosperm genotype) were scored as pigmented. The frequency of red and yellow families in the population corresponded to our expectation of 0.125 and 0.375, respectively (χ^2^ = 1.23, df = 1, *P*‐value = 0.27; χ^2^ = 0.18, df = 1, *P*‐value = 0.67). Running SNP‐based GWA, we identified lead hits at 11 kb from *P1* (SNP 1_48412077) and 40 kb from *Y1* (SNP 6_85019028), for red and yellow pigmentation, respectively (Fig. 2b; Tables S8, S9). We then ran QTL models fitting the eight parental alleles, again recovering major peaks closely linked to *P1* and *Y1* candidate loci (Fig. 2b). Estimation of parental allele effects identified REV as the donor of the red pigmentation, and MUS and NAL as the strongest yellow donors, in line with our expectation (Fig. 2c,d). Overall, analysis of kernel pigmentation provided validation of genotyping, the genetic map and inference of founder ancestry across the MEMA family genomes.

### The MexMAGIC founders capture an elevational cline in tassel branch number

Having mapped kernel pigmentation in the MexMAGIC, we next sought to characterize the genetic architecture underlying a well‐established elevational cline in tassel (male inflorescence) branch number (TBN). Native Mexican highland maize produces large tassels with few or no lateral branches, while the tassels of lowland varieties are highly branched (up to 40 branches), resembling the maize ancestor *parviglumis* (Fig. 3a; Wellhausen *et al*., 1952; Eagles & Lothrop, 1994; Sanchez *et al*., 2000; Perez‐Limón *et al*., 2022). A prior study of phenotypic and genetic diversity in native maize varieties (HiLo panel; Figs 3b, S9; Table S10) reported an excess of Qst/Fst associated with TBN between lowland and highland Mexican maize, consistent with locally acting selection (Janzen *et al*., 2022). We characterized the population structure of this previously studied HiLo panel and found a strong correlation with both elevation and TBN (Figs S10, S11), illustrating a fundamental challenge to the identification of underlying loci using GEA. In this context, the reduced population structure in the MexMAGIC presents an advantage. We evaluated the eight MexMAGIC founder varieties in a common garden and found that they followed the TBN cline with respect to source elevation (Fig. 3a), indicating the population to be suitable for investigation of the underlying genetic architecture. Specifically, we aimed to use the MexMAGIC to evaluate evidence for (1) clinal allelic series for TBN and (2) bias in the directionality of genome‐wide allele effects on TBN with respect to source elevation.

**Fig. 3:**
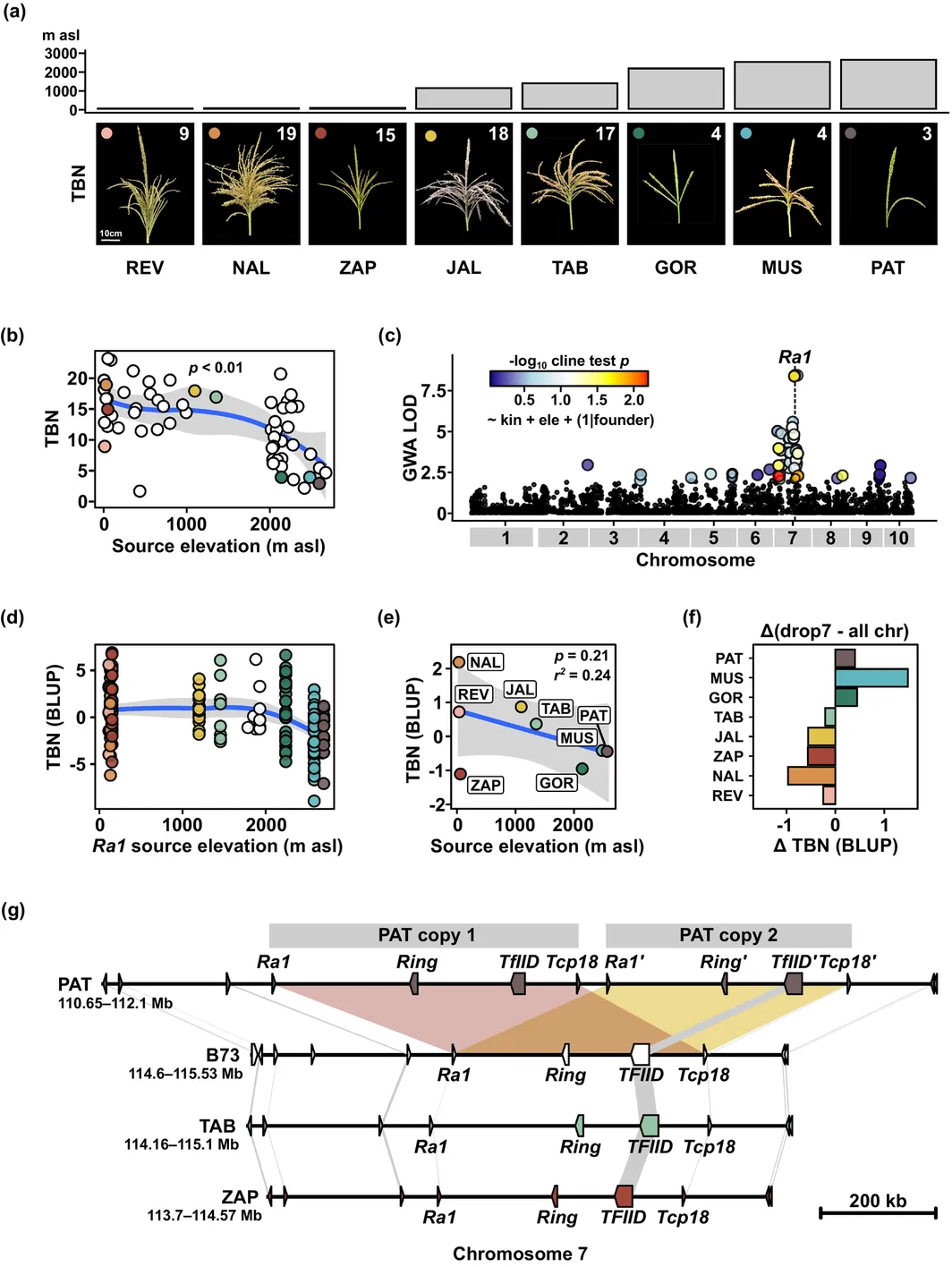
Tassel branch number follows an elevational cline in Mexican native maize. (a) Representative tassels of the eight MexMAGIC maize (*Zea mays* L. ssp. *mays*) founder accessions. The inbred founders REV, TAB and ZAP are here represented by related open‐pollinated accessions. Accessions are arranged by ascending source elevation (m asl, m above sea level) from left to right. Bars above the image show source elevation. The median tassel branch number (TBN) for each accession is shown in the upper right of the image. Colored points in the upper left of the images correspond to the color legend used in other panels. (b) Relationship between source elevation and TBN for the HiLo panel (open points) and the MexMAGIC founders (points colored as (a)). Blue line shows a LOESS fit, with the area extending ±1 SE. Model statistic refers to a linear fit. (c) Genome‐wide association (GWA) support (LOD) in the MexMAGIC (160 families) for association with TBN across the 10 maize chromosomes. All markers with LOD > 2 are shown. A reduced random sampling of lower‐supported markers is shown. Markers with LOD > 2 are colored by support for a second model (shown) fitting allele source elevation to TBN. The physical position of the candidate gene *Ra1* is indicated by a dashed vertical line. (d) Relationship between allele source elevation at *Ra1* and TBN for the MexMAGIC families. Source elevation was estimated as the product of genotype probabilities at *Ra1* and the founder elevations. Points are colored by the founder hard‐call at *Ra1* as in (a). Uncolored points represent families for which the founder call at *Ra1* was ambiguous. Blue line shows a LOESS fit, with the area extending ±1 SE. (e) Correlation between observed TBN and genomic BLUPs predicted by fitting marker effects from the MexMAGIC families to the founder haplotypes. Blue line shows a linear fit, with the area extending ±1 SE. Statistics shown for Pearson correlation. (f) Difference in predicted TBN for each founder between a model using all marker effects and a model dropping all chr 7 markers, including the *Ra1* QTL. (g) Microsynteny visualization of a genome duplication in PAT around the *Ra1* gene. The duplicated region spans *c*. 1.1 MB (chr 7: 114.6–115.53 MB in B73 ref v5) and encompasses 4 gene models including *Ra1*.

### Allele effects at a major QTL align with the elevational cline in tassel branch number

To characterize the genetic architecture of TBN in the MexMAGIC population, we evaluated 160 testcross families over 2 yr in a mid‐elevation common garden in western Mexico. We observed TBN values from 0 to 31, exceeding the range seen among the eight founder varieties and the HiLo panel (Table S7). The broad‐sense heritability of TBN was 0.62. GWA and the 8‐allele QTL model identified a major peak for TBN on chromosome (Chr) 7 (qTBN 7.1; hereafter, QTL are named using the prefix q, the trait abbreviation, the chromosome, and sequential numbering for each occurrence on the chromosome; Table S8) that co‐localized with the previously characterized TBN‐associated gene *Ramosa1* (*Ra1*: Fig. 3c; Sigmon & Vollbrecht, 2010; Gonzalez‐Segovia *et al*., 2019; Perez‐Limón *et al*., 2022; Strable *et al*., 2023). To test alignment between *Ra1* (followed here with marker 7_113574146) allele effects and the elevational cline, we applied a second model fitting allele source elevation to TBN, taking residual population structure into account and treating founder as a random effect. There was evidence (*P* = 0.025) of a negative correlation between source elevation of *Ra1* and TBN, driven largely by the negative effects of the highland MUS and PAT alleles (Figs 3c,d, S12–S14; Table S11). Interestingly, the GOR *Ra1* allele, from the highlands of northern Mexico, did not share the negative effects of MUS and PAT (Figs 3d, S13), although the GOR founder accession itself showed low TBN (Fig. 3a).

### A polygenic model recovers the elevational cline in TBN across founders

Although the genetic architecture of TBN was dominated by the QTL at *Ra1*, weaker signals were also found on other chromosomes (Fig. 3c; Table S9). Outside of the *Ra1* locus, top SNPs (LOD > 2) at a second peak on chr 7 (7_35152019) and another on chr 8 (8_160433628) also showed evidence of clinal allele effects, although others, including two (6_165825744 and 8_133594448) that co‐localized with previously reported TBN QTL (Xu *et al*., 2017), did not follow the elevational cline (Figs 3c, S14; Table S12). In the latter case, it was the mid‐elevation alleles that differed from both lowland and highland extremes. Variation along a cline may be driven by selection, by drift and demographic history, or by a combination of these factors, and, in itself, does not necessarily indicate local adaptation (Savolainen *et al*., 2013; Laroche & Lenormand, 2023). Building on GEA theory and the premise of the QTL sign test (Orr, 1998), we hypothesized that a shift from predominantly positive to predominantly negative genome‐wide allele effects across the cline would be consistent with directional selection on TBN. After dropping markers on chr 7, including the *Ra1* locus, SNP heritability for TBN remained at 0.33, indicating the presence of genome‐wide effects contributing to TBN variation that might be used to test the hypothesis of clinal allele distribution (Fig. S15). To assess bias in the distribution of genome‐wide effects with respect to the founder source, we used a polygenic model trained with the MexMAGIC families to predict TBN for the eight founder haplotypes. We found a positive correlation between observed and predicted founder TBN, and a concomitant negative correlation between predicted founder TBN and source elevation (Figs 3e, S16), indicating that the distribution of genome‐wide effects may indeed support the elevational cline. These correlations, however, were not statistically significant, largely due to poor prediction for ZAP. Furthermore, after removal of *Ra1* and other markers on chr 7, there was no evidence of a correlation between predicted TBN and source, MUS now also being poorly estimated, highlighting the limitations and sensitivity of our analysis due to the small number of founder alleles (Figs 3f, S17). The predicted difference between the TBN extremes NAL and PAT, however, was preserved in the absence of *Ra1*, lending some support to the hypotheses that loci outside the *Ra1* QTL show a bias in effect at either end of the elevation cline (Figs 3f, S17). In addition, TBN was predicted to be low in the northern highland GOR with or without inclusion of Chr 7 markers, consistent with the overall low TBN observed for this variety but the positive effect of the GOR *Ra1* allele.

### A duplication of *Ra1* in the genome of the highland variety PAT is linked to reduced TBN

To investigate possible causal variation in highland *Ra1*, we took advantage of whole‐genome assembly of a PAT haplotype sampled from the same accession as the PAT donor in the MexMAGIC, along with assemblies for the TAB (mid‐elevation) and ZAP (lowland) founders (www.maizegdb.org/HiLo_project). Microsynteny analysis of the *Ra1* locus across PAT, TAB, ZAP and the B73 reference revealed a *c*. 1.1 Mb duplication unique to PAT that contains four annotated gene models, including *Ra1* (Fig. 3g). The resulting two paralogous copies of *Ra1* in the PAT genome are 100% identical in their coding regions (Table S13). Comparison of PAT, TAB and ZAP *Ra1* models did not indicate major functional differences at the coding level (Table S14). Loss‐of‐function *ra1* mutants condition increased TBN (Vollbrecht *et al*., 2005; McSteen, 2006; Sigmon & Vollbrecht, 2010). We speculate that, conversely, an increase in *Ra1* dosage as a result of duplication in the PAT genome would reduce tassel branching. We generated and evaluated a B73xPAT BC_3_DH NIL, derived from the sequenced PAT haplotype and carrying PAT introgression at *Ra1*, and confirmed a reduction in TBN, providing further evidence to link *Ra1* duplication to low tassel branching (B73: TBN mean = 8.3; NIL: TBN mean = 4; Wilcoxon test: *P* < 0.0001; Fig. S18).

### Highland and lowland early flowering alleles segregate in the MexMAGIC population

Following TBN, we turned our attention to flowering time. In contrast to the single major QTL we found underlying TBN, prior studies lead us to anticipate that flowering variation would be driven by many smaller effect loci, offering more scope to address hypotheses related to the genetic architecture of clinal traits. The timing of flowering is crucial to maize local adaptation (Chardon *et al*., 2004). Well studied examples include photoperiod insensitivity in temperate regions (Salvi *et al*., 2007; Ducrocq *et al*., 2008; Bouchet *et al*., 2013; Yang *et al*., 2013; Huang *et al*., 2018) and accelerated – albeit chronologically slow – development in cool highland environments within the tropics (Mercer *et al*., 2008; Romero Navarro *et al*., 2017; Janzen *et al*., 2022). In addition, farmers consciously maintain both early and late maturing varieties that may be cultivated sequentially to ensure year‐round grain availability (Sanchez & Goodman, 1992; CONABIO, 2011) or planted simultaneously to account for climatic uncertainty (Wellhausen *et al*., 1952; Burgos‐May *et al*., 2004). Although temperate photoperiod insensitivity, highland developmental acceleration and farmer‐selected precocity can be characterized together as early flowering, it is unclear to what degree they are mechanistically equivalent or if they share a common genetic basis. In prior common garden evaluations across low, mid and high elevation sites in Mexico, the average flowering time (male flowering; days to anthesis, DTA) of the MexMAGIC founder varieties ranged from 60 to 91 d, the earliest being PAT, GOR (highland) and ZAP (lowland early maturity; Table S1; Ruíz Corral & Sánchez González, 1998), leading us to the expectation that both highland and lowland early flowering alleles segregate in the MexMAGIC population. As such, the MexMAGIC provides an opportunity to assess commonalities and convergence in selection for early flowering under distinct pressures.

We evaluated DTA in 160 MexMAGIC testcross families over 2 yr in a Mexican common garden field site. The broad‐sense and SNP heritabilities of DTA were 0.88 and 0.79, respectively, indicating a strong genetic component. We mapped DTA using the 8‐allele QTL model, merging the top 1% SNPs into 11 distinct QTL (Fig. 4a; Table S8). There was no consistent QTL‐wide trend in allele effect (here, we present effects accelerating flowering – i.e. reducing DTA – as positive) with respect to founder source (Figs 4b,c, S19). We did, however, observe QTL specific patterns, the QTL clustering broadly into groups driven by highland, mid‐elevation and lowland early flowering alleles (Fig. 4d). Applying the cline test used above for TBN, we found support (*P* < 0.1) for positive (i.e. greater flowering acceleration with increasing elevation) elevational clines at qDTA 8.1 and qDTA 8.2, and negative elevational clines at qDTA 2.3 and qDTA 9.3, indicative of distinct genetic architectures for highland and lowland precocity (Figs 4d, S19 and S20).

**Fig. 4:**
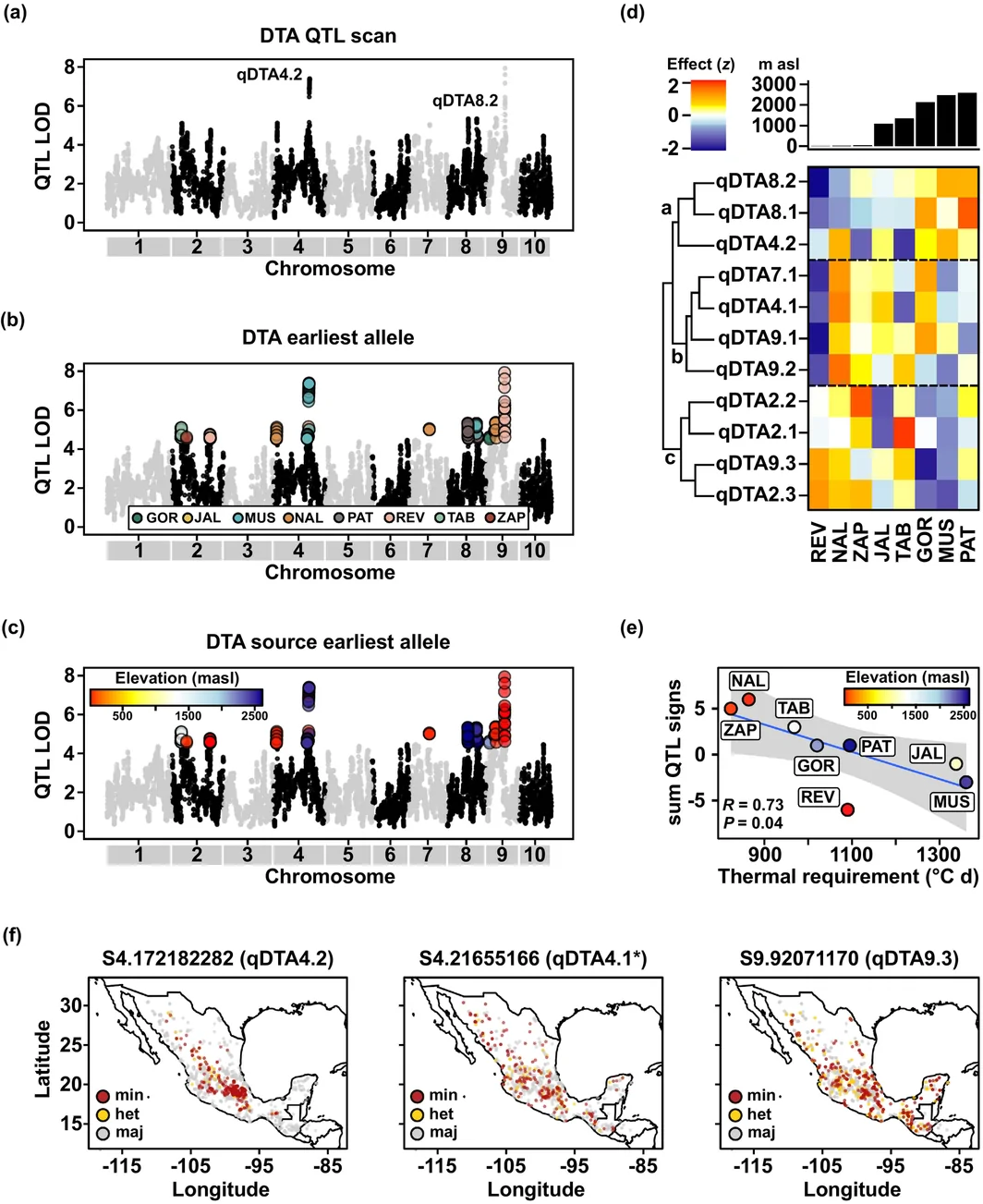
Highland and lowland early flowering alleles segregate in the MexMAGIC population. (a) Marker‐level support (LOD) in the maize (*Zea mays* L. ssp. *mays*) MexMAGIC (160 families) for association with flowering time (days to anthesis, DTA) across the 10 maize chromosomes. All markers with LOD > 4 are shown along with a reduced random sampling of lower‐supported markers. (b, c) As (a), with top (LOD > 4) markers colored to indicate the founder donating the earliest allele and the source elevation of the earliest allele, respectively. (d) Standardized allele effects (*z*) for each founder across DTA QTLs. Positive allele effects indicate flowering acceleration – that is, a reduction in DTA. Founders are arranged by elevation (bar plot; m asl, m above sea level). QTLs are clustered by allele effect, forming three major clusters labeled (a–c) for high‐, mid‐ and low‐elevation earliness. (e) Negative correlation between QTL effect sign sum and founder thermal requirement estimated by Ruíz Corral & Sánchez González (1998). The color of the dot represents the founder allele source elevation. (f) Genotype (maj – homozygous major allele; min – homozygous minor allele; het – homozygous heterozygote) in diverse Mexican maize of environmentally associated SNPs (CIMMYT panel) located within named MexMAGIC flowering QTL. The three QTL shown are illustrative of the three clusters shown in (d). * indicates that the marker corresponding to qDTA4.1 is located adjacent to, and not within, the QTL interval.

### Early flowering alleles assorted preferentially into founders with a lower thermal time requirement

Flowering can be expressed in terms of thermal time, the accumulated heat, rather than calendar days, providing a useful measure for comparison across environments (Tsimba *et al*., 2013). We obtained estimates of thermal time requirement for each founder variety from a previous report (Ruíz Corral & Sánchez González, 1998) and compared these to the complement of flowering alleles carried by each founder haplotype. Allele effects were categorized as positive (accelerating flowering), negative, or neutral, and the effect signs summed across the 11 QTL for each founder. A negative correlation (*r* ^2^ = 0.53, *P* < 0.05) was observed between previous estimates of thermal time flowering requirement for each founder variety (Ruíz Corral & Sánchez González, 1998) and the summed allele signs across the founder haplotypes (Fig. 4e). There was no significant relationship between either thermal time requirement or QTL sign sum with elevation, consistent with the presence of early and late‐flowering varieties within each elevational group and highlighting the importance of farmer selection. Indeed, although selection for accelerated flowering is a key component of highland adaptation (Eagles & Lothrop, 1994; Ruiz Corral *et al*., 2008), the lowest reported thermal time requirements and greatest accumulation of early flowering alleles among the MexMAGIC founders were found in the early maturing lowland varieties NAL and ZAP.

DTA QTL intervals contained a number of compelling candidate genes, including those with homology to flowering time regulatory components in other species, candidates identified in previous analyses of selection, and hits from environmental GWA mapping (Salvi *et al*., 2007; Guo *et al*., 2018; Table S15). Nonetheless, the resolution of our mapping and limited functional characterization of potential candidates precluded definitive identification of the genes underlying the QTL. That being said, we did observe overlap between qDTA8.2 and the well‐characterized flowering locus *Vegetative to generative transition 1* (*Vgt1*) (Salvi *et al*., 2007; Peiffer *et al*., 2014; Perez‐Limón *et al*., 2022), and between qDTA4.2 and the chromosomal inversion *Inv4m* that has been previously associated with early flowering in Mexican highland maize (Romero Navarro *et al*., 2017). To investigate evidence of our DTA variants contributing to adaptation across broader Mexican maize, we compared flowering QTL to SNP candidates obtained from environmental GWAS using the CIMMYT panel (Fig. 4f; Table S15; Li *et al*., 2025). Although no more than preliminary, it was intriguing to observe patterns of genetic stratification at environmentally associated SNPs that loosely mirrored the central highland or southern lowland origin of co‐localized early flowering alleles (Fig. 4e). Clearly, such an analysis would be best revisited in the future with full knowledge of the genes and variants underlying the DTA QTL.

## Discussion

To address hypotheses related to the genetic architecture of clinal traits, we mapped TBN and DTA in the MexMAGIC, a multi‐parent population designed to capture variation from across the range of Mexican native maize. The genetic architecture of TBN was dominated by a single large‐effect locus. By contrast, we mapped 11 QTL for DTA with evidence of a distinct genetic basis of accelerated flowering in highland and early maturing lowland varieties.

Cline theory predicts that the frequency of adaptive alleles will vary with respect to changes in selective pressures across the landscape (Huxley, 1938; Haldane, 1948; Fisher, 1950). GEA inverts this expectation to infer that alleles observed to change frequency over the environment are potentially adaptive (Lasky *et al*., 2023). Although the sampling in the MexMAGIC allows only limited inference regarding allele frequency in the founder varieties, it is possible to determine to what extent allele effects are concordant with their source environment and any observed phenotypic cline. Allele effects at the TBN candidate locus *Ra1* aligned well with the elevational cline in TBN, with the two alleles sourced from the highlands of central Mexico (MUS and PAT) showing strong negative effects on TBN (Fig. 3d). Theoretically, large effect alleles in a simple genetic architecture, such as the highland *Ra1*, are more likely to align to a cline than smaller effects (Le Corre & Kremer, 2012), and, consequently, to be detectable through GEA (Lasky *et al*., 2023). Nonetheless, the strong correlation between TBN and population structure observed in the HiLo panel (Fig. S11) would likely complicate detection of *Ra1* by GEA (Ahrens *et al*., 2021; Lasky *et al*., 2023). Indeed, we did not detect a significant association at *Ra1* in environmental GWAS using the CIMMYT panel (Table S15). In the context of the MexMAGIC, however, the correlation between source environment and *Ra1* allele effect on TBN survived correction for residual population structure, demonstrating the utility of the MAGIC approach.

*Ra1* encodes a plant‐specific C2H2 zinc finger transcription factor expressed near the base of inflorescence branch meristems to impose spikelet pair or short branch identity during ear and tassel development, respectively (Vollbrecht *et al*., 2005; McSteen, 2006; Sigmon & Vollbrecht, 2010). The timing of *Ra1* expression impacts the degree of determinacy and resulting inflorescence architecture (Strable *et al*., 2023). Loss‐of‐function *ra1* alleles in maize result in increased tassel branching (Vollbrecht *et al*., 2005; McSteen, 2006). Similarly, weak *Ra1* function in the maize ancestor *Z. mays* ssp. *parviglumis* is associated with highly branched tassels (Sigmon & Vollbrecht, 2010). By analogy, we hypothesize that reduced tassel branching in Mexican highland maize is driven by an increase in *Ra1* function. The duplication of *Ra1* in a Palomero Toluqueño genome assembly (a sister haplotype to the PAT MexMAGIC founder) is clearly suggestive of a potential mechanism for increased *Ra1* activity. The adaptive significance of reduced tassel branching in the Mexican highlands has not been determined, although we note that highland plants are tall and the tassels produce large quantities of pollen despite reduced branching (https://doi.org/10.6084/m9.figshare.c.8160125), potentially promoting outcrossing. Although effects at *Ra1* dominated our mapping results, we did find evidence of genome‐wide signals supporting the TBN cline, indicative of persistent directional selection. Furthermore, although the GOR founder haplotype sourced from the highlands of northern Mexico did not carry a reduced branching *Ra1* allele, the GOR variety itself di show low tassel branching, suggesting phenotypic convergence built on a distinct genetic architecture and offering further support for adaptive significance. Such speculation would be greatly strengthened by determining *Ra1* allelic diversity and frequency within the outbred varieties from which the MexMAGIC founders were sampled and more broadly in Mexican maize.

Maize flowering time is a heritable, complex trait governed by multiple small‐effect QTL segregating across the genome (Chardon *et al*., 2004; Flint‐Garcia *et al*., 2005; Buckler *et al*., 2009). Well‐defined latitudinal (Salvi *et al*., 2007; Ducrocq *et al*., 2008; Bouchet *et al*., 2013; Yang *et al*., 2013; Huang *et al*., 2018) and elevational (Mercer *et al*., 2008; Romero Navarro *et al*., 2017; Janzen *et al*., 2022) clines have been described in maize flowering time, blending differences in both photoperiod sensitivity and thermal requirement. Such variation is most clearly observed in a common garden (Mercer *et al*., 2008; Romero Navarro *et al*., 2017; Janzen *et al*., 2022), while the actual chronological flowering time in the native environment is strongly influenced by prevailing conditions. Beyond fulfilling the necessity of completing the cycle, maize flowering is further shaped by farmer selection for distinct maturities appropriate to different management strategies (Sanchez & Goodman, 1992; Wellhausen *et al*., 1952; Burgos‐May *et al*., 2004; CONABIO, 2011). The MexMAGIC segregates early‐flowering alleles contributed by both highland and lowland early maturing founders. The pattern of allelic effects we observed suggests limited molecular parallelism between accelerated flowering in highland and early maturing lowland varieties, although we acknowledge the limits of our sampling. In this context, major early flowering alleles on chromosome 8 (here and Romero Navarro *et al*., 2017; Janzen *et al*., 2022; Perez‐Limón *et al*., 2022) suggest flowering in Mexican highland maize to have more in common with early flowering in North American Flint and Northern European varieties (Ducrocq *et al*., 2008) than with early maturing Mexican lowland varieties.

The QTL qDTA4.2 co‐localized with the previously described chromosomal inversion *Inv4m* that has been shown to segregate at high frequency in both Mexican highland maize and the Mexican highland teosinte *Zea mays* L. spp. *mexicana* Iltis & Doebley (hereafter, *mexicana*; Pyhäjärvi *et al*., 2013; Crow *et al*., 2020; Calfee *et al*., 2021). The highland inverted form of *Inv4m* has been associated with a three‐day acceleration of flowering time in native maize (Romero Navarro *et al*., 2017). Although we linked the highland MUS and PAT alleles at qDTA4.2 to early flowering in our mid‐elevation common garden, we saw similarly strong effects for the mid‐elevation TAB and low‐elevation NAL alleles. Although the Palomero Toluqueño genome assembly (a sister haplotype to the PAT MexMAGIC founder) contains the inverted allele at *Inv4m* and we found evidence that PAT and MUS founders carry *Inv4m* as the result of gene flow from *mexicana* teosinte (Fig. S21; Hufford *et al*., 2013; Gonzalez‐Segovia *et al*., 2019), there is no expectation that the lowland NAL would carry *Inv4m*, while the TAB founder genome assembly definitively contains the standard form. Collectively, our observations indicate that multiple functionally early alleles at qDTA4.2 are present in Mexican native maize, linked to both inverted and standard forms of *Inv4m*. As such, the association of early flowering with *Inv4m* may be incidental – albeit with potential consequences for subsequent selection, in contrast to a previously described example in *Mimulus* in which an inversion *per se* was the proximal cause of linked flowering acceleration (Twyford & Friedman, 2015).

In contrast to the behavior of a large‐effect allele, such as the highland *Ra1*, more complex genetic architectures incorporating a larger number of small allele effects exert less pressure on any given locus to align with a phenotypic cline, even if the clinal effect itself is strong (Lotterhos, 2023). Alongside *Ra1*, we detected evidence of small‐effect QTL associated with TBN, including those that were supported by co‐localization with previously reported loci (Xu *et al*., 2017). The effects associated with minor QTL for TBN, for the most part, did not align with the elevational cline, consistent with theoretical expectation. Small‐effect loci likely still contribute to a phenotypic cline under the condition that the balance of positive and negative alleles carried by any given individual is appropriate to its location. Whole‐genome prediction of TBN for the MexMAGIC founder haplotypes lent some support to such a model, with the cline being partially maintained even after removal of strong effects at *Ra1*. In outbred populations, such as native maize varieties, a complex genetic architecture not only predicts weak patterns of changing allele frequency over a cline but would also accommodate genetic variation between phenotypically similar individuals in any specific location, provided the overall allelic complement was functionally equivalent. The flowering loci identified in the MexMAGIC showed a similar behavior as the TBN minor QTL with little discernible pattern in allele effects over the source environment. The apparently distinct genetic architectures of highland and lowland early flowering in the MexMAGIC further complicated efforts at simple alignment of allele effects to a single environmental axis. Nonetheless, whole‐genome prediction based on founder haplotypes was consistent with previously reported thermal time requirement for the founder varieties.

The clinal traits we have characterized in the MexMAGIC potentially contribute to local adaptation. The genetic architecture of local adaptation itself, however, including all the components that define ‘fitness’ for a crop species, is necessarily far more complex. Taking into account the possibility of plasticity, pleiotropy and epistatic interactions, the challenge to GEA approaches is clear. Further evaluation of resources such as the MexMAGIC, notably in different environments, will provide a valuable complement to GEA and genetic differentiation methods in efforts to understand the basis of local adaptation with a view to conservation and responsible use of important biodiversity resources.

## Competing interests

None declared.

## Author contributions

RJHS, RR‐A and CSG designed the research; MNS‐V, ALA‐N, MRR‐F, GCC‐M, JC‐M, JVT‐R, SP‐L, CSG, RR‐A and RJHS generated and evaluated the MexMAGIC population; MGP, OHSP and JSS collected and analyzed additional field data; MRR‐F and PY performed genotypic analysis; VL and KAF performed genome assembly; FL, DER and JR‐I performed environmental association analysis; SP‐L, RJHS, PY, DER and JR‐I conducted primary analysis and interpretation of results; all authors contributed to writing the manuscript.

## Disclaimer

The New Phytologist Foundation remains neutral with regard to jurisdictional claims in maps and in any institutional affiliations.

## Supporting information


**Fig. S1** Early generation ears.
**Fig. S2** Four‐way crosses.
**Fig. S3** Intermating generations.
**Fig. S4** Genetic characterization of the MexMAGIC families: parental haplotypes, population structure, parental assignment in MEMA families.
**Fig. S5** Outlying families in genetic PCo1.
**Fig. S6** Genetic characterization of the MexMAGIC families: population structure by chromosome.
**Fig. S7** Field evaluation of the MexMAGIC testcross population.
**Fig. S8** Field evaluation Ameca 2022.
**Fig. S9** Sampling of Mexican native varieties reported in Janzen *et al*. (2022).
**Fig. S10** Population structure in the Mexican HiLo panel.
**Fig. S11** Population structure by elevation in the Mexican HiLo panel.
**Fig. S12** GWAS for tassel branch number in the MexMAGIC.
**Fig. S13** Allele effects at *Ra1*.
**Fig. S14** Tassel branch number allele effects with respect to source elevation.
**Fig. S15** Genome‐wide marker effects on tassel branch number.
**Fig. S16** Genome‐wide prediction of tassel branch number.
**Fig. S17** Impact of dropping chromosome 7 markers on predicted tassel branch number.
**Fig. S18** Tassel branching in a B73‐*Ra1*‐PT BC_3_DH NIL.
**Fig. S19** Effect against founder elevation at the lead SNP for the 11 QTLs identified for days‐to‐anthesis in the MexMAGIC.
**Fig. S20** Days‐to‐anthesis for MexMAGIC families against founder source elevation at flowering QTL.
**Fig. S21** *Zea mays* sp. *mexicana* introgression at *Inv4m*.
**Methods S1** Description of population generation and evaluation.

**Table S1** List of founder varieties, accession numbers, source location and additional trait data.
**Table S2** Environmental descriptors for source locations of Mexican native maize varieties characterized in Romero Navarro *et al*. (2017) and MexMAGIC founders (best CIMMYT match for derived inbreds).
**Table S3** Contribution of environmental descriptors to environmental PCs.
**Table S4** Proportion of missing and heterozygous sites in the chip genotyping dataset.
**Table S5** Principal coordinate analysis of MexMAGIC family genotype data.
**Table S6** Projected parents of origin for gene models based on hard‐called ancestry for each MexMAGIC family.
**Table S7** BLUPs and fitted values estimated for traits scored in MexMAGIC field evaluation.
**Table S8** QTL position and effect.
**Table S9** Effect estimates for founder alleles at each QTL.
**Table S10** Accessions described in Janzen *et al*. (2022).
**Table S11** Genotype probabilities at Ra1 (7_113571427) and tassel branch number for MexMAGIC families; includes source environment data for founder hard call.
**Table S12** Model output for fits of tassel branch number and days‐to‐anthesis on allele source environment.
**Table S13** B73 and PT gene models at *Ra1*.
**Table S14** Top gene hits from MEGALMM environmental GWAS.
Please note: Wiley is not responsible for the content or functionality of any Supporting Information supplied by the authors. Any queries (other than missing material) should be directed to the *New Phytologist* Central Office.

## Data Availability

Data that support the findings of this study are openly available in the https://doi.org/10.6084/m9.figshare.c.8160125 (1. Pipeline for genetic mapping on the MexMAGIC population: code and objects required to perform a minimal example of SNP‐association (GWAS) and QTL mapping; 2. Palomero Toluqueño tassel shedding pollen.) Genome accession nos. PAT, ERS16235157; GCA_963555555; TAB, ERS16997546; GCA_964199785; ZAP, ERS16997547; GCA_964199775.
